# Transcriptome analysis of two pepper genotypes infected with pepper mild mottle virus

**DOI:** 10.3389/fgene.2023.1164730

**Published:** 2023-04-20

**Authors:** Ziming Zhang, Xiaofan Chang, Shuangxia Luo, Yanhua Wang, Shuxin Xuan, Jianjun Zhao, Shuxing Shen, Wei Ma, Xueping Chen

**Affiliations:** Collaborative Innovation Center of Vegetable Industry in Hebei, College of Horticulture, Hebei Agricultural University, Baoding, China

**Keywords:** pepper, PMMoV, transcriptome, mechanism, genotype

## Abstract

Pepper mild mottle virus (PMMoV) poses a significant threat to pepper production because it is highly contagious and extremely persistent in soil. Despite this threat, little is known about the molecular processes that underlie plant responses to pepper mild mottle virus. Here, we performed RNA sequencing of tolerant (“17-p63”) and susceptible (“16-217”) pepper genotypes after pepper mild mottle virus or mock inoculation. Viral accumulation in systemic leaves was lower in the pepper mild mottle virus-resistant 17-p63 genotype than in the pepper mild mottle virus-sensitive 16-217 genotype, and infection symptoms were more apparent in systemic leaves of 16-217 than in those of 17-p63 at the same timepoints during the infection process. We identified 2,959 and 2,159 differentially expressed genes (DEGs) in systemic leaves of infected 16-217 and 17-p63, respectively. Through Gene Ontology (GO) and Kyoto Encyclopedia of Genes and Genomes (KEGG) enrichment analysis of differentially expressed genes from both genotypes revealed significant enrichment of the MAPK signaling pathway, plant–pathogen interaction, and flavonoid biosynthesis. A number of differentially expressed genes showed opposite trends in relation to stress resistance and disease defense in the two genotypes. We also performed weighted gene co-expression network analysis (WGCNA) of all samples and identified modules associated with resistance to pepper mild mottle virus, as well as seven hub genes. These results identify candidate virus resistance genes and provide insight into pepper defense mechanisms against pepper mild mottle virus.

## Introduction

Pepper (*Capsicum annuum* L.) is an annual or perennial plant of the genus *Capsicum* in the family *Solanaceae*. Its fruit is rich in vitamin C and also has a unique pungent flavor, making it both an important vegetable crop and an economically valuable condiment. Furthermore, the capsaicin found in pepper fruits is an important chemical material (Ashrafi et al., 2012). Viral infection has a significant impact on pepper yield and quality, and 45 plant viruses are known to be capable of infecting peppers (Lockhart and Fischer, 1974; Wetter et al., 1984; Kenyon et al., 2014). Among these viruses, pepper mild mottle virus (PMMoV) causes particularly yield losses during pepper cultivation because of its high transmission ability (mainly through soil and seeds) and its specific site of disease (fruit). Northeast China reported that 50% of peppers in the region were infected with PMMoV, resulting in a one-third yield loss in 2014 (Li et al., 2016).

PMMoV is a positive-sense single-stranded RNA virus of the tobacco mosaic virus genus. Its genome contains 6356–6357 nucleotides and encodes at least four proteins: the 126-kDa and 183-kDa replication-associated proteins, movement protein, and coat protein (Velasco, 2002). PMMoV-like damage was documented in the United States as early as 1964 (Greenleaf et al., 1964), although the virus was not named until 1984 (Wetter et al., 1984). PMMoV infection is currently one of the key factors affecting pepper production worldwide. In the early stage of PMMoV infection, pepper plants show slight leaf wrinkling and mosaicism, followed by production of fruits with uneven discoloration and mottled symptoms, some with brown necrotic streaks (Wang et al., 2006). PMMoV can also cause human disease symptoms such as fever and abdominal pain (Colson et al., 2010).

Viral infection of plants is a complex process involving multiple interactions between the host plant and the invading virus. During the initial stage of viral infection, host plants activate signaling cascades upon recognition of the invading virus and inhibit virus replication and propagation by altering various metabolic and signal transduction pathways (Jiao et al., 2020). Nonetheless, how the host plant recognizes the virus and the defense mechanisms by which it controls virus infection are not yet known. Four alleles, *L* ^*1*^, *L* ^*2*^, *L* ^*3*^, and *L* ^*4*^, are associated with tobamovirus resistance in pepper genotypes, and the *L* gene is one of the most effective resistance genes against *Tobamovirus* spp., (Holmes, 1937). The *L* gene contains an NBS-LRR structural domain, and each *L* gene allele encodes a different protein. The LRR domain, especially in the N-terminal LRR region, is important for the interaction of *L* genes with viral coat proteins (Tomita, 2011). Disease resistance associated with the *L* gene in pepper is often accompanied by a hypersensitive response or overexpression of numerous disease-resistance-related genes, resulting in local necrosis. The mechanism by which *L* genes confer against resistance *Tobamovirus* spp., is similar to that of plant ETI. The most effective method of PMMoV resistance is still the breeding of pepper varieties that contain the *L* gene, but PMMoV is gradually overcoming *L* gene resistance, and some researchers have reported P_1,2,3,4_ pathogenic PMMoV that can overcome *L* ^*1*^, *L* ^*2*^, *L* ^*3*^, and *L* ^*4*^ (Genda et al., 2007; Antignus et al., 2008). Unfortunately, no new resistance genes against PMMoV have been found. Therefore, understanding the defense mechanism of pepper against PMMoV infection and discovering novel disease resistance genes have become a focus of current research.

Because of the complexity of host plant–virus interactions, which involve multiple physiological processes, transcriptome analysis has become a key step for studying the mechanisms of such interactions (Lu et al., 2012; Muthamilarasan and Prasad, 2013). Observation of changes in gene expression during PMMoV infection can help us to characterize the defense mechanisms of pepper against PMMoV. Previous transcriptomic research on PMMoV-infected pepper leaves revealed that cellular autophagy had a role in PMMoV resistance (Jiao et al., 2020), and another study demonstrated inhibition of the photosynthetic process when leaves were infected by PMMoV (Kalapos et al., 2021). However, there has not yet been a global comparison of gene expression in systemic leaves of PMMoV-susceptible and PMMoV-tolerant genotypes during infection.

**FIGURE 1:**
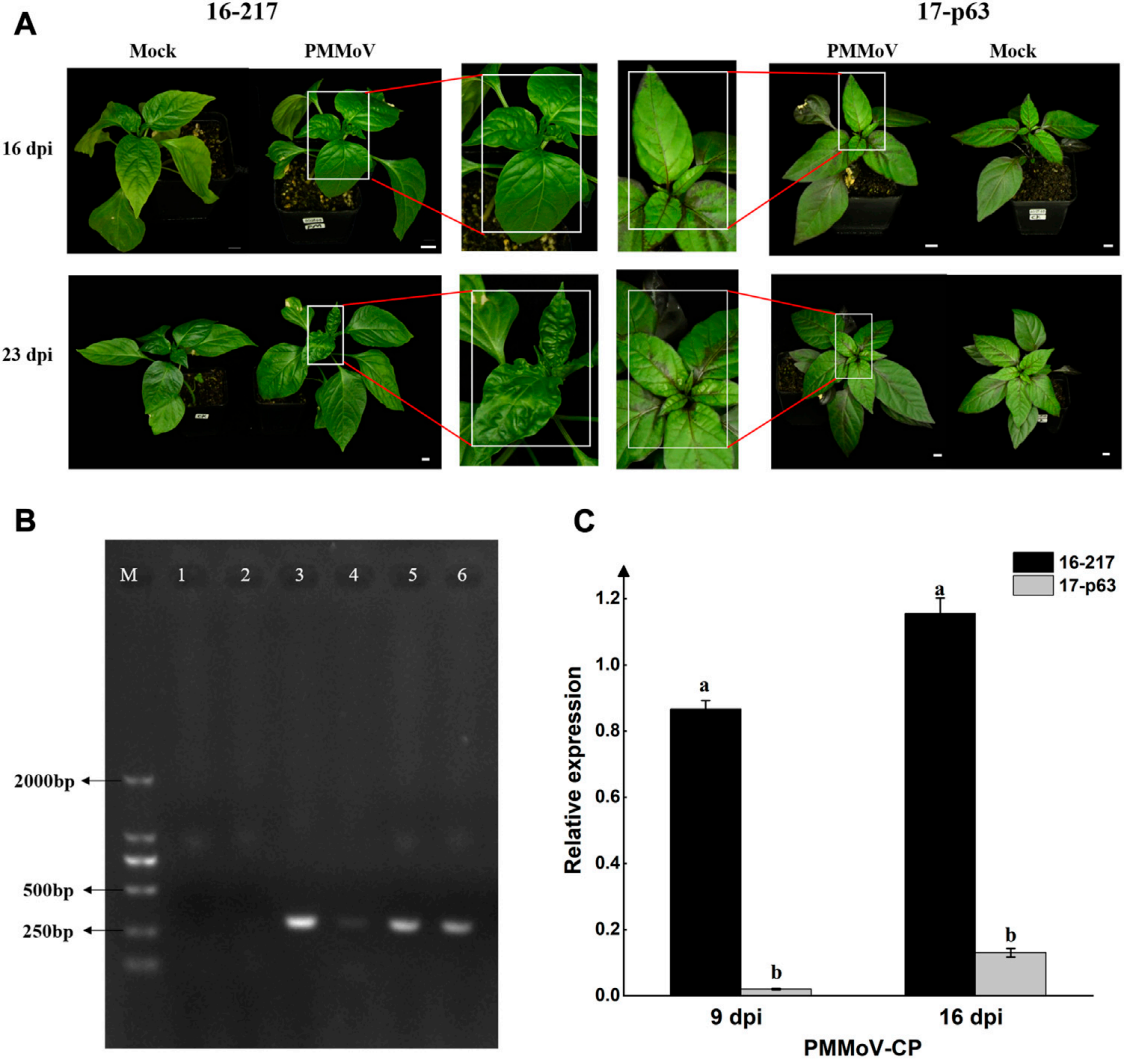
Symptoms of Pepper mild mottle virus (PMMoV) infection and accumulation of PMMoV coat protein in upper leaves of 16-217 and 17-p63. **(A)** Symptoms of PMMoV in systemic leaves of 16-217 and 17-p63 at 16 and 23 days post inoculation (dpi) (bar = 1 cm). **(B)** Semiquantitative RT-PCR for the PMMoV coat protein gene in systemic leaves at 9 dpi. M: DL2000 marker; 1: mock-inoculated 17-p63; 2: mock-inoculated 16-217; 3 and 4: PMMoV-inoculated 17-p63; 5 and 6: PMMoV-inoculated 16-217. **(C)** qRT-PCR of the PMMoV coat protein gene in both genotypes at 9 and 16 dpi. All values are presented as the mean ± SD (*n* = 3). Different lowercase letters indicate significant differences between means (*p* < 0.05).

Pepper 17-p63 is a PMMoV-infection-tolerant genotype identified in the field. Interestingly, 17-p63 differs from PMMoV-resistant genotypes containing the *L* gene in that it can be infected with PMMoV but show no significant symptoms (Figure 1A). In this study, WGCNA analysis was combined with transcriptome sequencing to study the transcriptomic responses of 17-p63 (a tolerant genotype) and 16-217 (a susceptible genotype) to PMMoV infection. We identified differentially expressed genes (DEGs) in systemic leaf samples of PMMoV- and mock-inoculated pepper plants at 9 and 16 days after inoculation and constructed a gene co-expression network. Genes involved in flavonoid biosynthesis, plant–pathogen interaction, and MAPK signaling were differentially expressed in the two genotypes in response to infection, and hub genes related to PMMoV resistance were identified by WGCNA. Our study provides insights into the molecular mechanisms of resistance to PMMoV infection in pepper and contributes to our understanding of plant–virus interactions.

## Results

### Disease symptoms in PMMoV-infected pepper plants and virus detection

In comparison to mock-inoculated plants, the PMMoV-inoculated 16-217 plants displayed distortion of systemic leaves by 16 days post inoculation (dpi) and more severe symptoms by 23 dpi (Figure 1A). However, systemic leaves of 17-p63 appeared normal at both time points (Figure 1A), indicating that this genotype had a higher level of tolerance to PMMoV.

To ensure that PMMoV inoculation was successful, we used semiquantitative reverse transcription PCR (RT-PCR) to detect the presence of the PMMoV coat protein in virus- and mock-inoculated plants. The presence of PMMoV was confirmed in PMMoV-inoculated plants of both genotypes but not in the mock-inoculated plants (Figure 1B). PMMoV virions were first detected on upper systemic leaves at 9 dpi in both genotypes (Supplementary Figure S2), and expression of the PMMoV coat protein (CP) gene was much higher in the upper leaf of 16-217 than that of 17-p63 at 9 and 16 dpi (Figure 1C), indicating that PMMoV accumulation was lower in the systemically infected leaves of 17-p63.

### Genes differentially expressed at two time points during PMMoV infection

PMMoV virions were first detected on upper systemic leaves at 9 dpi in both genotypes; PMMoV-inoculated 16-217 plants displayed distortion of systemic leaves by 16 dpi, whereas 17-p63 plants showed no symptoms. To explore the mechanisms by which the two genotypes responded to PMMoV infection, we constructed 24 RNA libraries from systemic leaves of PMMoV- and mock-inoculated plants of both genotypes at 9 dpi and 16 dpi. In total, 146.85 Gb of valid reads were acquired from these libraries (Table 1), over 94% of which could be mapped to the pepper reference genome. Over 97% of the valid reads from each library had a quality score of Q30 (sequencing error rate less than 0.1%), and the GC content ranged from 42.5% to 43% among samples (Table 1).

**TABLE 1:** Statistics describing the Illumina sequencing data.

Sample	Raw data		Valid data		Valid ratio	Q20%	Q30%	GC content%
	Read	Base (G)	Read	Base (G)	(Reads)			
PL9a	44700010	6.71	42150404	6.32	94.3	99.96	97.19	42.5
PL9b	42179800	6.33	39786292	5.97	94.33	99.97	97.03	42.5
PL9c	43706976	6.56	41572940	6.24	95.12	99.96	97.19	42.5
PL16a	44084094	6.61	41590608	6.24	94.34	99.97	97.69	43
PL16b	41781632	6.27	39565642	5.93	94.7	99.97	97.65	43
PL16c	44382686	6.66	42175148	6.33	95.03	99.97	97.69	43
ML9a	41531428	6.23	39274518	5.89	94.57	99.97	97.28	43
ML9b	44262046	6.64	42005406	6.30	94.9	99.97	97.52	43
ML9c	43543584	6.53	41362058	6.20	94.99	99.97	97.55	43
ML16a	42509954	6.38	40750868	6.11	95.86	99.97	97.53	42.5
ML16b	45249688	6.79	43636200	6.55	96.43	99.97	97.46	42.5
ML16c	42634398	6.40	40618620	6.09	95.27	99.98	97.54	43
PZ9a	42885074	6.43	40552596	6.08	94.56	99.97	97.31	42.5
PZ9b	42504396	6.38	40357440	6.05	94.95	99.95	97.05	42.5
PZ9c	40101394	6.02	37812228	5.67	94.29	99.97	97.29	42.5
PZ16a	40074276	6.01	37830760	5.67	94.4	99.97	97.43	42.5
PZ16b	43862550	6.58	41407488	6.21	94.4	99.97	97.37	42.5
PZ16c	44928936	6.74	42043830	6.31	93.58	99.96	97.31	42.5
MZ9a	43435794	6.52	40372086	6.06	92.95	99.98	97.08	43
MZ9b	43442210	6.52	41045120	6.16	94.48	99.98	97.16	43
MZ9c	41899896	6.28	39687006	5.95	94.72	99.96	97.02	42.5
MZ16a	42642542	6.40	40883156	6.13	95.87	99.96	97.4	42.5
MZ16b	42574612	6.39	40630682	6.09	95.43	99.97	97.51	42.5
MZ16c	43799060	6.57	41985296	6.30	95.86	99.97	97.51	42.5

**FIGURE 2:**
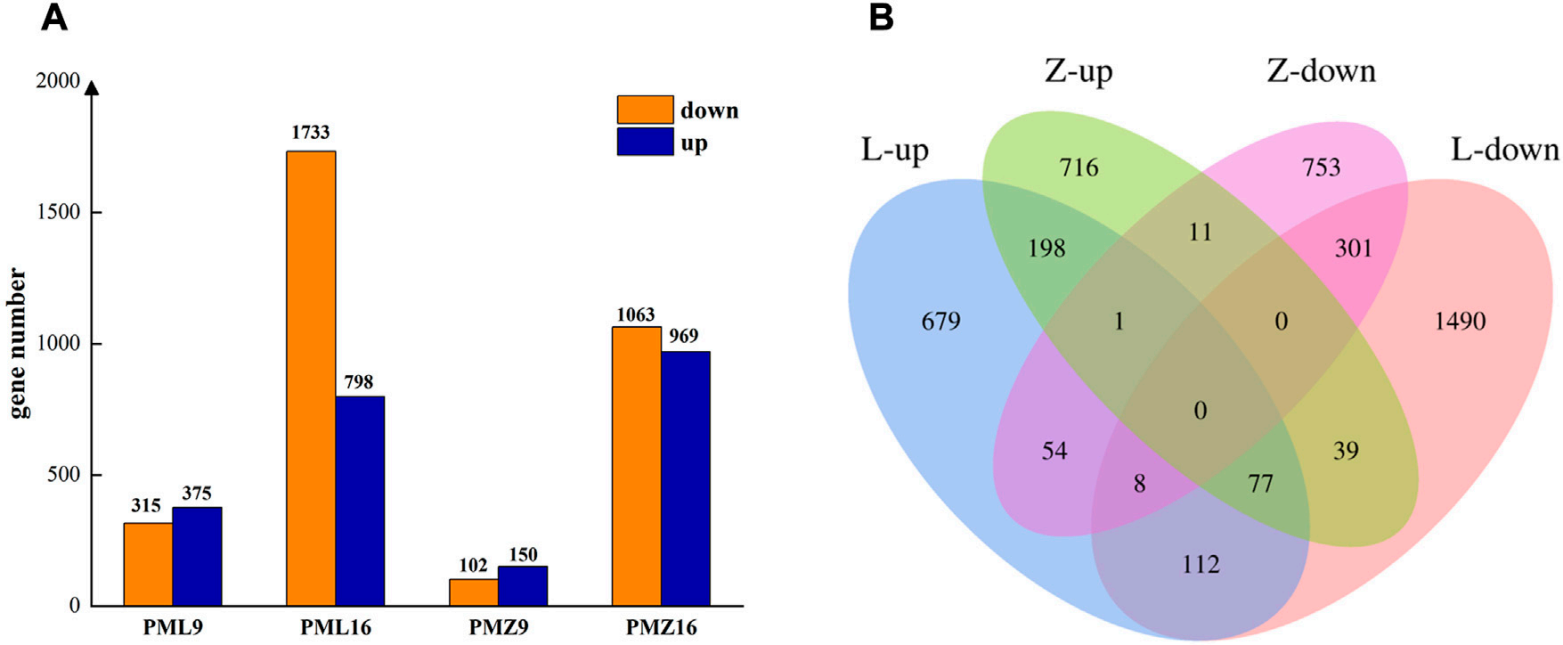
Differentially expressed genes in 16-217 and 17-p63. **(A)** Numbers of up- and downregulated genes. PML9 and PML16 represent comparisons between PMMoV- and mock inoculated 16-217 plants at 9 dpi and 16 dpi. PMZ9 and PMZ16 represent comparisons between PMMoV- and mock-inoculated 17-p63 plants at 9 dpi and 16 dpi. **(B)** Venn diagram of up- and downregulated genes. L, 16-217 genotype. Z, 17-p63 genotype.

Differentially expressed genes (DEGs) were identified by comparing gene expression between PMMoV-inoculated and mock-inoculated samples using the criteria (q < 0.05 and |log_2_FC| ≥ 1). There were 4439 DEGs in total between the PMMoV- and mock-inoculated plants across all time points and genotypes. The numbers of upregulated and downregulated DEGs increased between 9 and 16 dpi in both genotypes, and there were more DEGs in susceptible 16-217 than in tolerance 17-p63 at both stages of infection (Figure 2A). Across both time points, 276 upregulated DEGs and 309 downregulated DEGs were common to both genotypes (Figure 2B). However, 93 genes displayed contrast responses to PMMoV infection in the two genotypes: 39 were upregulated in 17-p63 and downregulated in 16-217, whereas 54 were upregulated in 16-217 and downregulated in 17-p63. Overall, infections had less effect on gene expression in 17-p63 than in 16-217.

### Functional enrichment analysis of DEGs shared between both genotypes

**FIGURE 3:**
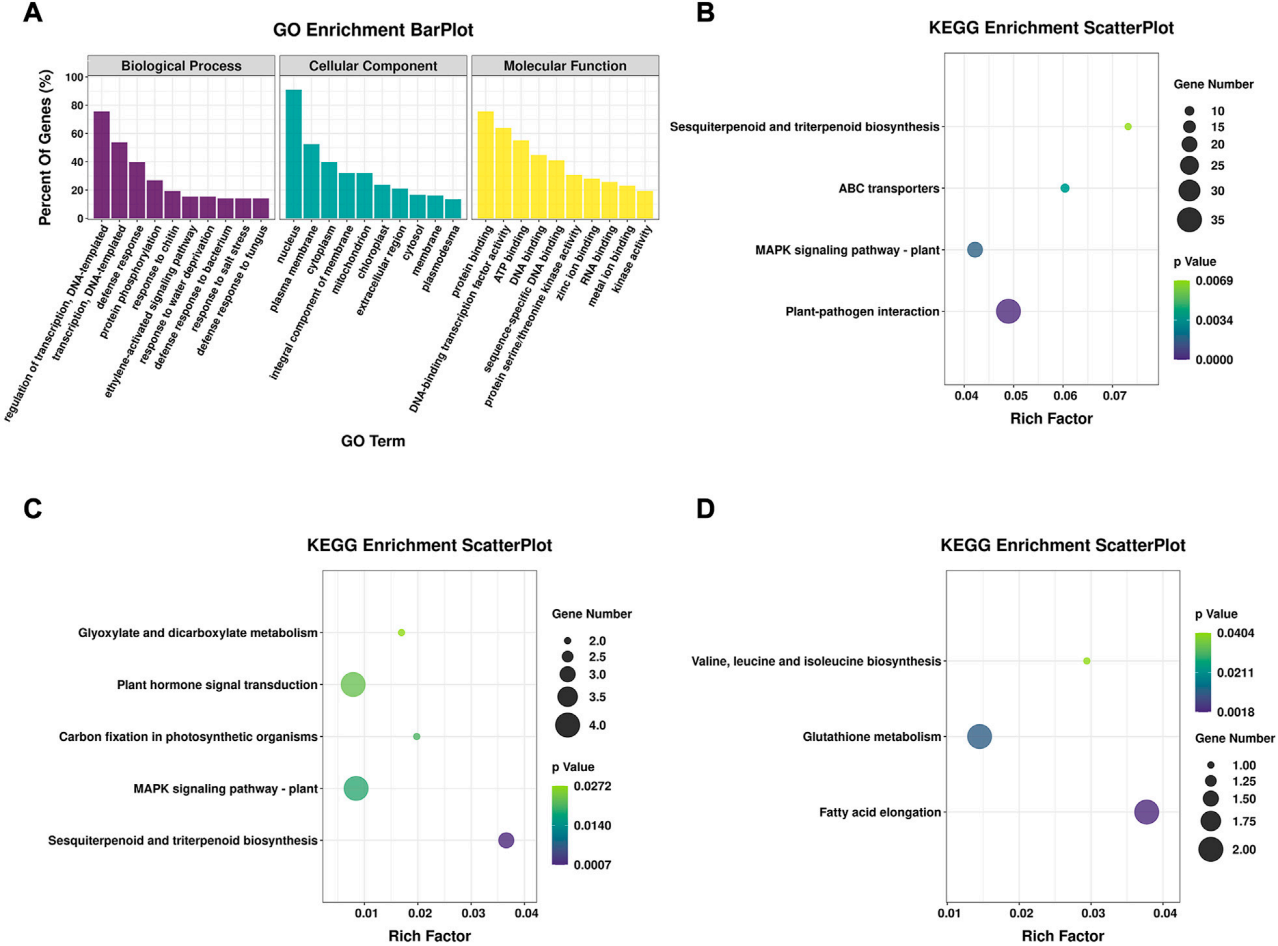
GO and KEGG enrichment of DEGs. **(A)** GO enrichment analysis of shared DEGs from both genotypes, showing the top 10 terms in three categories. **(B)** Significantly enriched KEGG pathways of shared DEGs from both genotypes. **(C)** Significantly enriched KEGG pathways of 54 genes that were upregulated in 16-217 but downregulated in 17-p63. **(D)** Significantly enriched KEGG pathways of 39 genes that were upregulated in 17-p63 and downregulated in 16-217.

The main functions of the DEGs were clarified using GO and KEGG enrichment analyses. In total, 678 DEGs were shared between 16-217 and 17-p63 during PMMoV infection (Figure 2B), 533 of which were annotated with at least one GO term or KEGG pathway. Results show that GO terms enriched in the shared DEGs included the biological process terms regulation of transcription, transcription, defense response, and protein phosphorylation; the cellular component terms nucleus and plasma membrane; and the molecular function terms protein binding, DNA-binding transcription factor activity, and ATP binding (Figure 3A).

Among the significantly enriched KEGG pathways were plant-pathogen interaction, MAPK signaling pathway-plant, ABC transporters, and sesquiterpenoid and triterpenoid biosynthesis (Figure 3B). A number of these enriched pathways were consistent with an important role for secondary metabolites and fundamental immunity in the defense of pepper against PMMoV infection.

Fifty-four DEGs were upregulated in susceptible 16-217 but downregulated in tolerant 17-p63, whereas 39 DEGs showed the opposite pattern (Figure 2B; Supplementary Table S1). We therefore performed KEGG enrichment analysis on these two DEG sets. The 54 genes that were upregulated in 16-217 and downregulated in 17-p63 were most significantly enriched in sesquiterpenoid and triterpenoid biosynthesis, MAPK signaling pathway-plant, carbon fixation in photosynthetic organisms, plant hormone signal transduction, and glyoxylate and dicarboxylate metabolism (Figure 3C). The 39 genes that were upregulated in 17-p63 but downregulated in 16-217 were most significantly enriched in fatty acid elongation, glutathione metabolism pathways, and valine, leucine and isoleucine biosynthesis (Figure 3D). Thus the two genotypes appeared to differ in aspects of their response to PMMoV infection, including terpene, hormone, and fatty acid synthesis and glutathione metabolism.

### Functional enrichment analysis of specific DEGs

**FIGURE 4:**
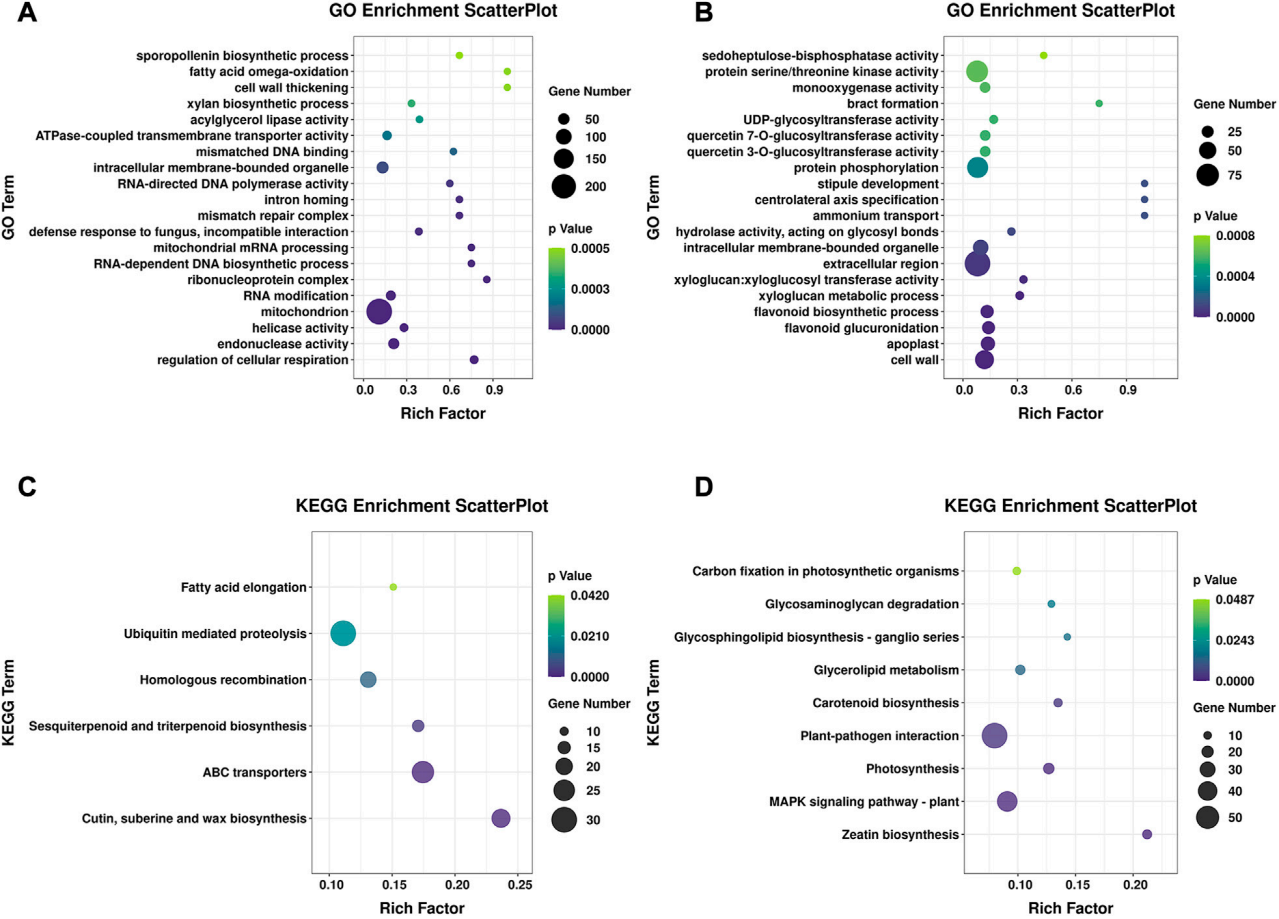
GO and KEGG enrichment of specific DEGs. **(A)** GO enrichment of specific DEGs in 16-217, showing the top 20 GO terms. **(B)** GO enrichment of specific DEGs in 17-p63, showing the top 20 GO terms. **(C)** Significantly enriched KEGG pathways of specific DEGs in 16-217. **(D)** Significantly enriched KEGG pathways of specific DEGs in 17-p63.

We next performed an enrichment analysis of DEGs specific to the susceptible genotype 16-217 and the tolerant genotype 17-p63. Among the DEGs we identified, 2281 were differentially expressed only in 16-217 and 1480 only in 17-p63. The top five GO terms enriched in 16-217-specific DEGs were regulation of cellular respiration, endonuclease activity, helicase activity, mitochondrion, and RNA modification (Figure 4A), whereas the top five GO terms enriched in 17-p63-specific DEGs were cell wall, apoplast, flavonoid glucuronidation, flavonoid biosynthetic process, and xyloglucan metabolic process (Figure 4B). In the KEGG enrichment results, 16-217-specific DEGs were most significantly enriched in cutin, suberin and wax biosynthesis, ABC transporters, sesquiterpenoid and triterpenoid biosynthesis, homologous recombination, ubiquitin-mediated proteolysis, and fatty acid elongation (Figure 4C). The 17-p63-specific DEGs were most significantly enriched in zeatin biosynthesis, MAPK signaling pathway-plant, photosynthesis, plant-pathogen interaction, and carotenoid biosynthesis, glycerolipid metabolism, glycosphingolipid biosynthesis, glycosaminoglycan degradation, and carbon fixation in photosynthetic organisms (Figure 4D).

### Identification of DEGs involved in plant MAPK signaling pathways

Through KEGG enrichment results of DEGs shared between both genotypes highlighted the main pathways of pepper response to PMMoV. Four pathways were significantly enriched, including Mitogen-activated protein kinase (MAPK) signaling pathways (Figure 4A). MAPK signaling pathways play a pivotal role in perception of pathogen attack and initiation of downstream defenses (Meng and Zhang, 2013). Transcriptome analysis indicated that 63 and 65 DEGs were involved in MAPK pathways in 17-p63 and 16-217, respectively (Supplementary Table S2), 20 of which were differentially expressed in both genotypes.

Among these 20 DEGs, four were predicted to encode receptors that identify pathogen infection (*Capana01g001931*, *Capana02g000535*, *Capana04g001610*, *Capana05g000276*); one (*Capana02g000535*) was downregulated in the resistant genotype and upregulated in the susceptible genotype at 16 dpi. The other three were upregulated after infection with PMMoV. Four genes were annotated as mitogen-activated protein kinase 4/5 (*Capana02g001328*, *Capana03g003283*, *Capana03g003841*, *Capana06g000135*); they were downregulated in the resistant genotype and upregulated in the susceptible genotype after infection with PMMoV. Three genes predicted to encode WRKY transcription factors were upregulated in both genotypes at 16 dpi (*Capana06g001506*, *Capana08g000429*, *Capana09g001251*). These genes play a regulatory role in the camalexin synthesis pathway. Two genes (*Capana02g002477*, *Capana02g002478*) upregulated in both genotypes at 16 dpi were predicted to encode acidic endochitinase. *Capana01g002771* was annotated as serine/threonine-protein kinase OXI1 and was upregulated in both genotypes at 16 dpi. *Capana02g003359* was annotated as a calcium-binding protein, it was upregulated at both time points in the resistant genotype, but downregulated at 9 dpi and upregulated at 16 dpi in the susceptible genotype. *Capana03g003881* was annotated as phosphate transporter PHO1 and was downregulated in both genotypes at 16 dpi. *Capana07g000998* was annotated as the transcription factor bHLH041; it was upregulated in both genotypes at 16 dpi, but downregulated in the susceptible genotype and no significant changed in the resistant genotype at 9 dpi. *Capana07g002306* was annotated as mitogen-activated protein kinase kinase 2-like; it was upregulated only at 9 dpi in the resistant genotype and upregulated only at 16 dpi in the susceptible genotype. The last gene was annotated as a late blight resistance protein homolog; it was upregulated only at 16 dpi in the resistant genotype, but upregulated at 9 dpi and downregulated at 16 dpi in the susceptible genotype. On the basis of the identifies and expression trends of these 20 shared DEGs, we hypothesized that a MAPK cascade was likely to activate the first layer of plant immunity after recognition of PMMoV infection.

### Identifications of DEGs involved in plant–pathogen interaction

The plant–pathogen interaction KEGG pathway was significantly enriched in the DEGs shared between both genotypes. We identified 100 and 92 DEGs associated with the plant–pathogen interaction pathway (ko04626) in 16-217 and 17-p63, respectively (Supplementary Table S3), 30 of which were shared by both genotypes. These genes are likely to have important roles in the pepper–PMMoV interaction. Based on their FPKM values (greater than 10 in at least one sample), we identified 8 DEGs that may have a particularly important role in plant–pathogen interaction. Five were predicted to encode WRKY transcription factors or receptor proteins (Capana01g001931, Capana03g002635, Capana05g001632, Capana06g001506, and Capana10g001548) and were upregulated in both genotypes after PMMoV inoculation. Two were involved in Ca^2+^ signal transduction (Capana02g003359 and Capana03g000955) and were upregulated in tolerant 17-p63 but downregulated in susceptible 16-217. *Capana03g003841*, which is predicted to encode a light-dependent short hypocotyls 4-like protein, was downregulated in 17-p63 at 9 and 16 dpi but upregulated in 16-217 at 9 dpi.

### Identification of DEGs involved in flavonoid biosynthesis

Plants synthesize a variety of secondary metabolites that function in plant protection (Zaynab et al., 2018), and several secondary metabolite pathways were significantly enriched in the DEGs (Figures 4A, B). Interestingly, the biological process GO term flavonoid biosynthetic process was enriched in the 17-p63-specific DEGs but not in the 16-217-specific DEGs.

The flavonoid biosynthetic process (ko00941) KEGG pathway was enriched in both genotypes in response to PMMoV infection. Twenty-nine DEGs were identified as being related to flavonoid biosynthetic process in the two genotypes: 13 DEGs in 17-p63 and 20 DEGs in 16-217 (Supplementary Table S4). Four were common to both genotypes: two were downregulated after infection (Capana09g001507 and Capana12g000351), and two showed opposite responses to infection in the two genotypes. *Capana03g001713* was upregulated in tolerant 17-p63 but downregulated in susceptible 16-217 at 16 dpi and was predicted to encode dihydroflavonol 4-reductase (DFR), a key enzyme in the anthocyanidin biosynthetic pathway. *Capana12g000351* was upregulated in 17-p63 at 16 dpi but downregulated in 16-217 at 9 dpi and was predicted to encode chalcone synthase (CHS), a central enzyme of flavonoid biosynthesis. These results suggest that flavonoid metabolites play an important part in resistance of pepper to PMMoV infection.

### WGCNA identified candidate modules associated with PMMoV tolerance

**FIGURE 5:**
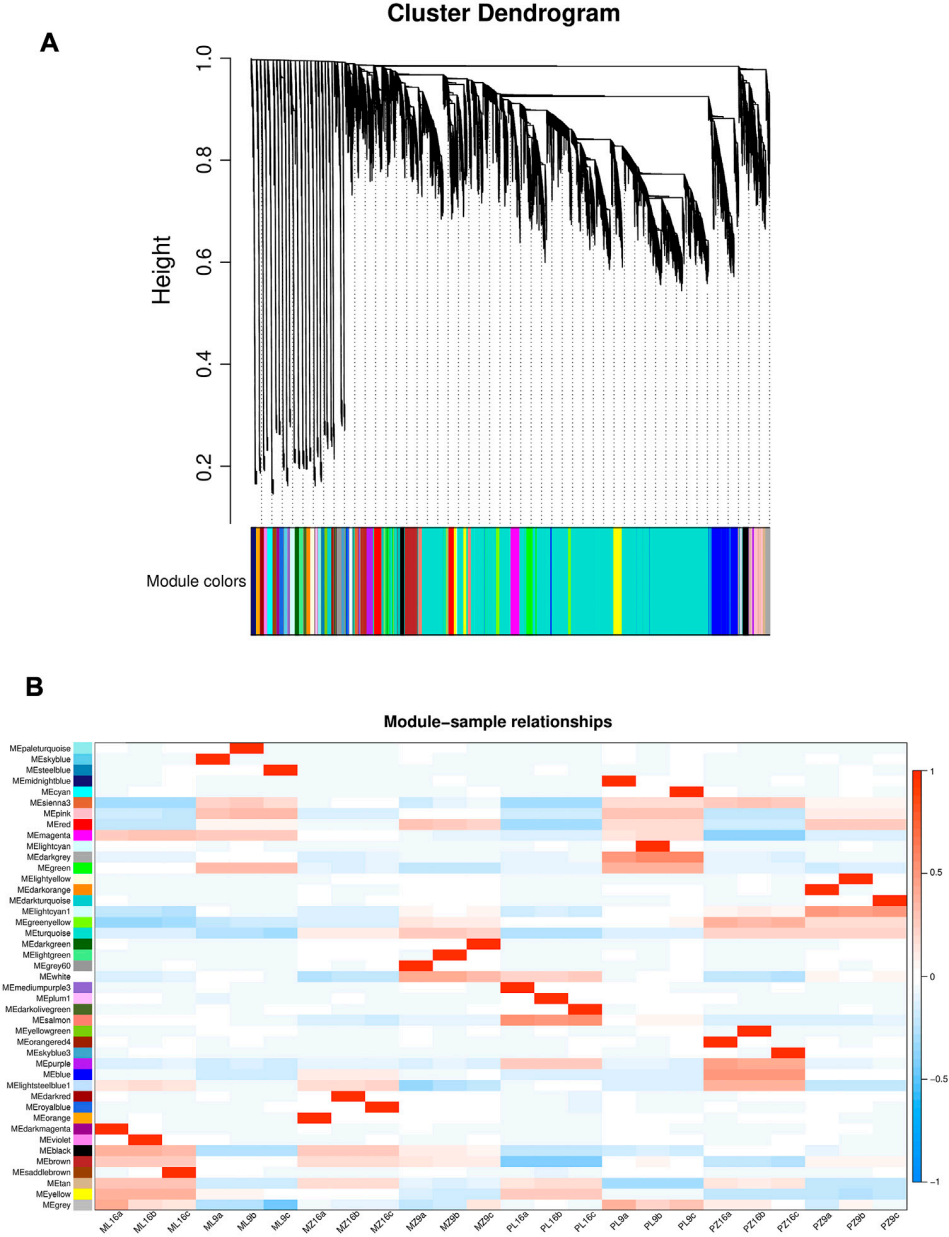
Weighted gene co-expression network in 17-p63 and 16-217. **(A)** Module-level clustering diagram. Different colors represent different modules. **(B)** Correlations of sample traits with WGCNA modules. Rows correspond to modules, and columns correspond to 24 samples. P, PMMoV-inoculated. M, mock-inoculated. L, 16-217 genotype. Z, 17-p63 genotype. 9, 9 dpi. 16, 16 dpi. a, b, and c represent three biological replicates. For example, PML9 represents the samples of pepper 16-217 after 9 days of PMMoV infection. Cell color indicates the value of the correlation coefficient between a module and a sample: red represents a positive correlation and blue represents a negative correlation.

We removed genes with very low or no expression (FPKM < 0.5) in all samples, leaving 27,794 genes for WGCNA analysis (Langfelder and Horvath, 2008). A co-expression network was built based on pairwise correlations of gene expression in all samples, and modules were defined as clusters of highly correlated genes. We identified 43 modules that were correlated with different samples (Figure 5A). Two modules (lightcyan1 and blue) were highly correlated with PMMoV-infected samples of 17-p63 at 9 dpi and 16 dpi, respectively, and we focused on these modules to identify candidate PMMoV resistance genes (Figure 5B). The lightcyan1 module associated with infected 17-p63 at 9 dpi contained 70 genes, and the blue module associated with infected 17-p63 at 16 dpi contained 1,735 genes.

### Hub genes from candidate modules related to PMMoV tolerance

**FIGURE 6:**
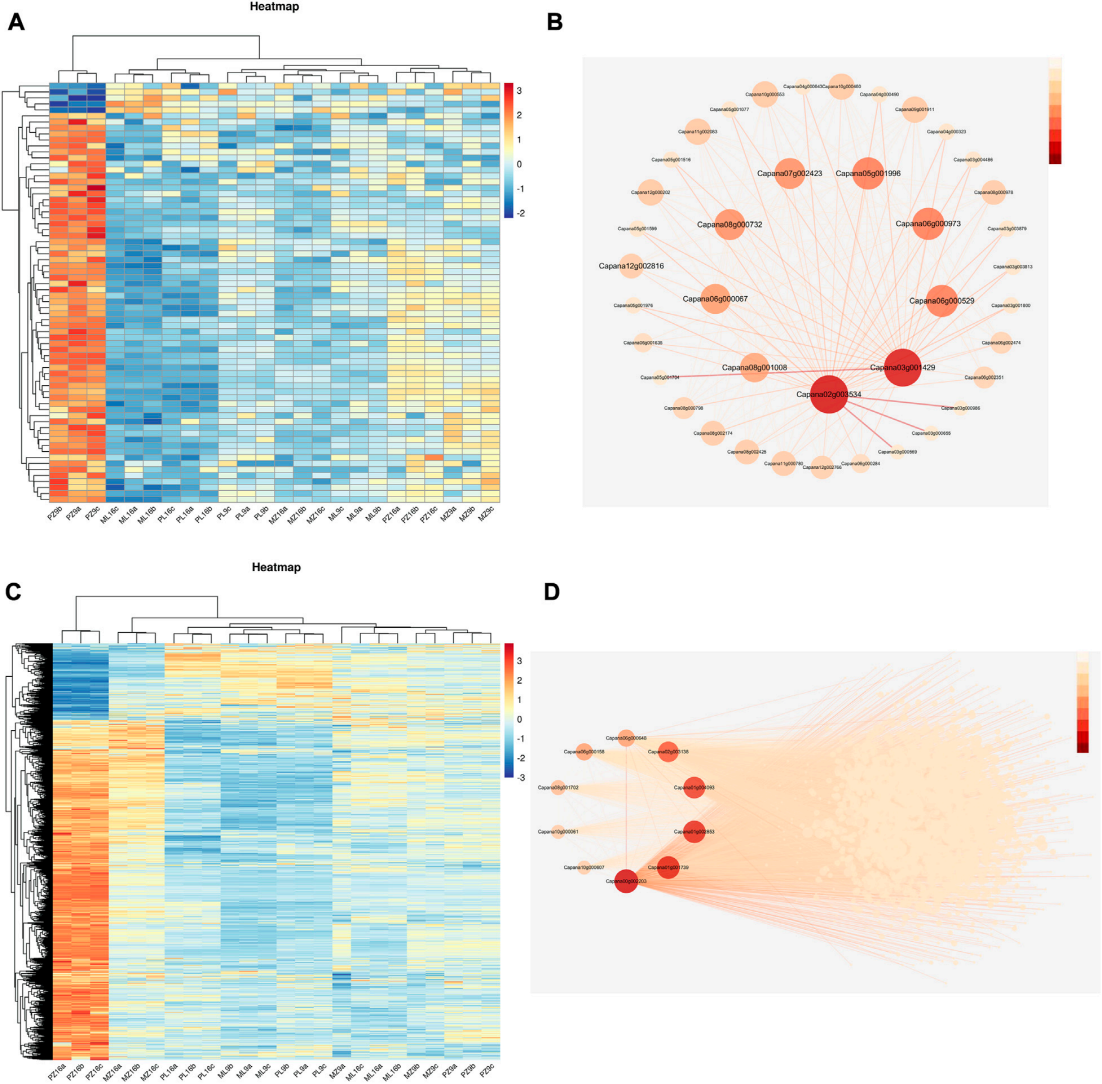
Heatmap of gene expression patterns and gene regulatory networks of the lightcyan1 and blue modules. **(A)** Heatmap of gene expression in the lightcyan1 module; log_2_(FPKM) is indicated by the color scale. **(B)** Regulatory network of the top 10 genes in the lightcyan1 module; the intensity of the red color indicates the magnitude of the connectivity value. **(C)** Heatmap of gene expression in the blue module; log_2_(FPKM) is indicated by the color scale. **(D)** Regulatory network of the top 10 genes in the blue module; the intensity of the red color indicates the magnitude of the connectivity value.

A heatmap showed that most genes in the lightcyan1 module were highly expressed only in PMMoV-infected 17-p63 at 9 dpi (Figure 6A), and 10 were identified as hub genes based on their high connectivity values. These hub genes were predicted to encode EID1-like F-box protein 3 (Capana06g000067), galactosyltransferase 5 (Capana02g003534), desiccation protectant protein Lea14 homolog (Capana08g001008), glycine-rich cell wall structural protein 1 (Capana05g001996), betaine aldehyde dehydrogenase 1 (Capana06g000973), BTB/POZ domain-containing protein At1g55760 (Capana07g002423), enhanced disease resistance 2-like isoform X1 (Capana06g000529), and three uncharacterized proteins (Capana08g000732, Capana12g002816, and Capana03g001429) (Figure 6B). Enhanced disease resistance 2-like isoform X1 is associated with disease resistance, and the other proteins have been associated with abiotic stress responses.

Most blue-module genes were highly expressed only in PMMoV-infected 17-p63 at 16 dpi (Figure 6C), and 10 were identified as hub genes based on their high connectivity values. These encoded acetylajmalan esterase-like (Capana01g004093), L-ascorbate oxidase (Capana02g003138), aspartic proteinase nepenthesin-2 (Capana06g00064), putative boron transporter 3 (Capana01g001739), GDSL esterase/lipase LTL1-like (Capana06g000158), receptor-like protein kinase THESEUS 1 (Capana10g000061), subtilisin-like protease SBT1.9 (Capana00g002203), nectarin-1-like (Capana08g001702), and two uncharacterized proteins (Figure 6D). Seven of these genes (Capana06g000529, Capana01g004093, Capana02g003138, Capana06g000648, Capana06g000158, Capana10g000061, and Capana00g002203) have documented functions related to pathogen resistance and may therefore have important roles in the tolerance of 17-p63 to PMMoV infection.

### Verification of differential gene expression by quantitative PCR

**FIGURE 7:**
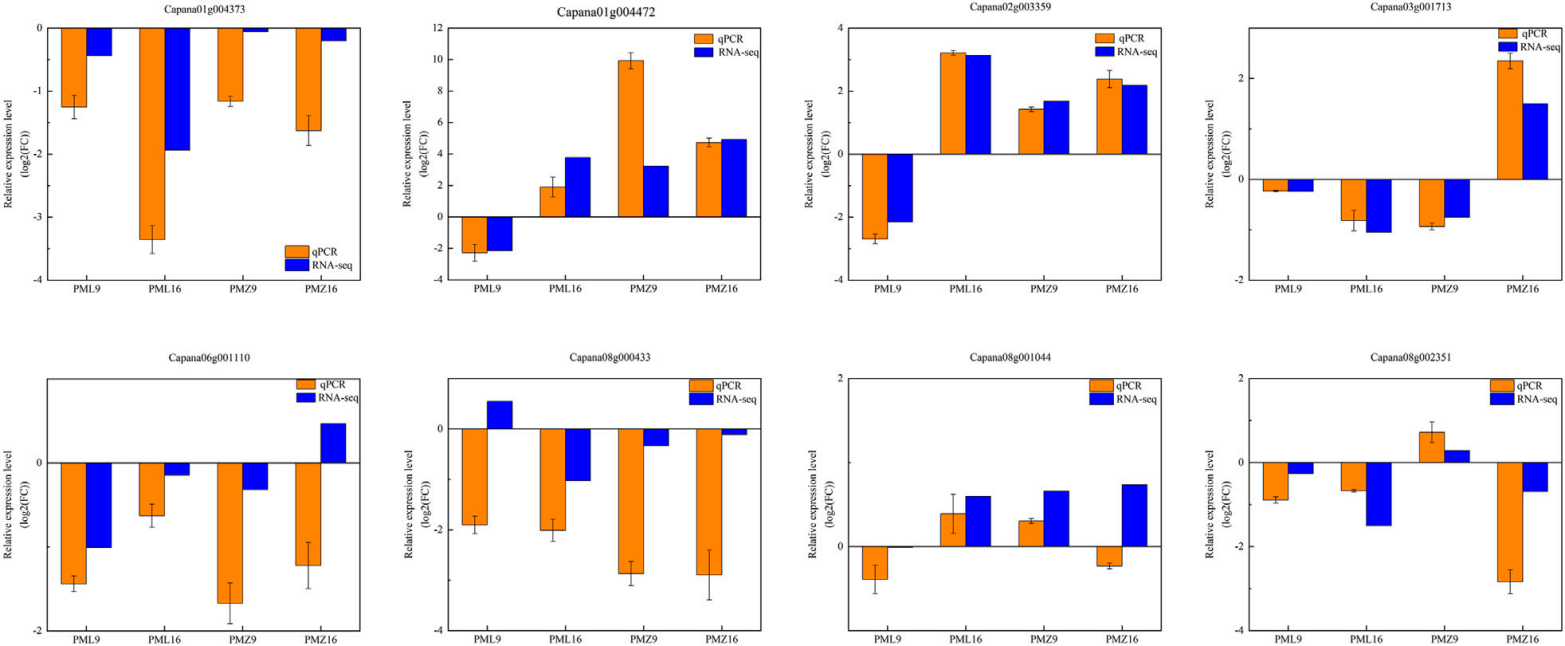
qRT-PCR expression validation for eight randomly selected genes. The beta tubulin gene was used as the reference gene.

To verify the RNA-seq results, eight DEGs were randomly selected for real-time quantitative reverse transcription PCR (qRT-PCR) analyses using specific primers (Supplementary Table S5). Three encoded WRKY transcription factors associated with the plant–pathogen interaction pathway, WRKY41 (Capana01g004472), WRKY53 (Capana08g001044), and WRKY40 (Capana06g001110); two encoded MAPK signaling pathway components, CML45 (Capana02g003359) and LRR receptor ERECTA (Capana08g000433); two encoded phenylpropanoid biosynthetic enzymes, CCOAOMT6 (Capana08g002351) and HCT (Capana01g004373); and one encoded a key enzyme of flavonoid biosynthesis, DFR (Capana03g001713). Expression fold changes measured by qRT-PCR and RNA-seq were similar (Figure 7).

## Discussion

PMMoV is an RNA virus that threatens pepper crops and causes economic losses worldwide (Shi et al., 2022). Viable viral infection depends on survival of the host plant (Wu et al., 2019), but the mechanisms by which plants combat viruses are not fully understood. In the present study, we used RNA-seq to compare gene expression changes in systemic leaves of PMMoV-infected pepper plants from a susceptible and a tolerant genotype. After PMMoV infection, there were no infection symptoms in the upper leaves of 17-p63, but the upper leaves of 16-217 showed clear infection symptoms (Figure 1A). Virus accumulation was also lower in the upper leaves of 17-p63 than in those of 16-217 at both time points (Figure 1C). These findings suggest that the ability of PMMoV to mount a systemic infection and to move to the upper leaves was repressed in 17-p63.

A previous study examined the transcriptional responses of susceptible pepper plants to PMMoV infection at one time point and identified 197 DEGs while GO enrichment analysis showed that these DEGs were mainly related to plant biological and abiotic stresses, and KEGG enrichment analysis showed enrichment in secondary metabolite biosynthesis and amino acid metabolism (Jiao et al., 2020). In the present work, we studied the transcriptomic responses of pepper plants to PMMoV infection using two genotypes (susceptible and tolerant) and two time points after PMMoV infection. The results enabled us to identify candidate genes related to PMMoV tolerance in pepper. Identification of the PMMoV tolerant genotype 17-p63 provides a foundation for understanding the specific mechanisms by which pepper resists PMMoV infection.

We identified 2,959 and 2,158 genes that were differentially expressed in response to PMMoV infection in the susceptible and tolerant genotypes, respectively, and these genes may therefore have a role in pepper resistance to PMMoV infection. Many of the DEGs were associated with signal transduction, defense response, secondary metabolite biosynthesis, and transcription. These results were consistent with a previous study in which PMMoV infection of susceptible peppers activated defense-response genes that could promote production of antimicrobial secondary metabolites to limit further spread of the virus (Jiao et al., 2020).

Both genotypes could be infected by PMMoV, but 16-217 showed obvious symptoms, whereas no significant symptoms were observed in 17-p63. It is possible that both genotypes have the same pattern-recognition receptors that respond to virus infection and that 17-p63 is asymptomatic because of the presence/activity of specific metabolic pathways that inhibit virus multiplication and transmission. From what has been mentioned above GO enrichment analysis revealed enrichment of the flavonoid biosynthesis pathway in the 17-p63-specific DEGs. Flavonoid compounds act as defenses against fungi and viruses (Cushnie and Lamb, 2005), and many studies have shown that flavonoid accumulation is an important mechanism by which plants resist pathogen infection (Dong and Lin, 2021). For example, expression of flavonoid biosynthetic genes was induced after inoculation of alfalfa with the fungal pathogen *Fusarium oxysporum* f. sp. *medicaginis*, and levels of multiple flavonoids increased (Gill et al., 2018). *Stylosanthes* spp., synthesized flavonoid compounds in response to *Colletotrichum* infection by upregulating expression of flavonoid synthesis-related genes (Jiang et al., 2021), and similar flavonoid biosynthesis genes respond to oomycete infection in the phylogenetically distant species *Marchantia polymorpha* (liverwort) and *Nicotiana benthamiana* (angiosperm) (Carella et al., 2019; Fernie, 2019). We also identified two DEGs that are closely associated with the synthesis of flavonoid compounds: Capana03g001713, which encodes anthocyanidin reductase (DFR), and Capana12g000351, which encodes chalcone synthase (CHS), the first committed enzyme in flavonoid biosynthesis. The DFR gene was upregulated in the resistant genotype and downregulated in the susceptible genotype at 16 dpi. CHL was downregulated in the resistant genotype at 16 dpi and upregulated in susceptible at 9 dpi. The difference in their expression between the two genotypes may be responsible for the difference in disease resistance. These results suggest that flavonoid compounds may play an important part in the resistance of 17-p63 to PMMoV infection. Nonetheless, how flavonoids could function in pepper resistance to PMMoV infection remains to be investigated.

Plant hormones are also important metabolites that function in abiotic stress responses. Changes in expression of genes related to plant hormone pathways suggested that PMMoV infection altered plant hormone signal transduction in both genotypes. Plant viruses can severely affect growth hormone signaling, thereby affecting normal plant growth, and four hormones primarily regulate plant defense against pathogens: salicylic acid, jasmonic acid, ethylene, and abscisic acid (An and Mou, 2011, Kazan and Lyons, 2014; Alazem and Lin, 2015; Klessig et al., 2018; Zhou and Zhang, 2020; Mullender et al., 2021). In the present study, we found many DEGs related to auxin synthesis. Among the 12 DEGs with FPKM > 10 in 16-217, 8 were annotated as auxin response factors. These eight DEGs were downregulated at 16 dpi in PMMoV-infected 16-217, but their expression was unchanged in 17-p63. The wrinkled leaf symptoms seen in 16-217 may be related to the effect of PMMoV on the auxin signaling pathway.

**FIGURE 8:**
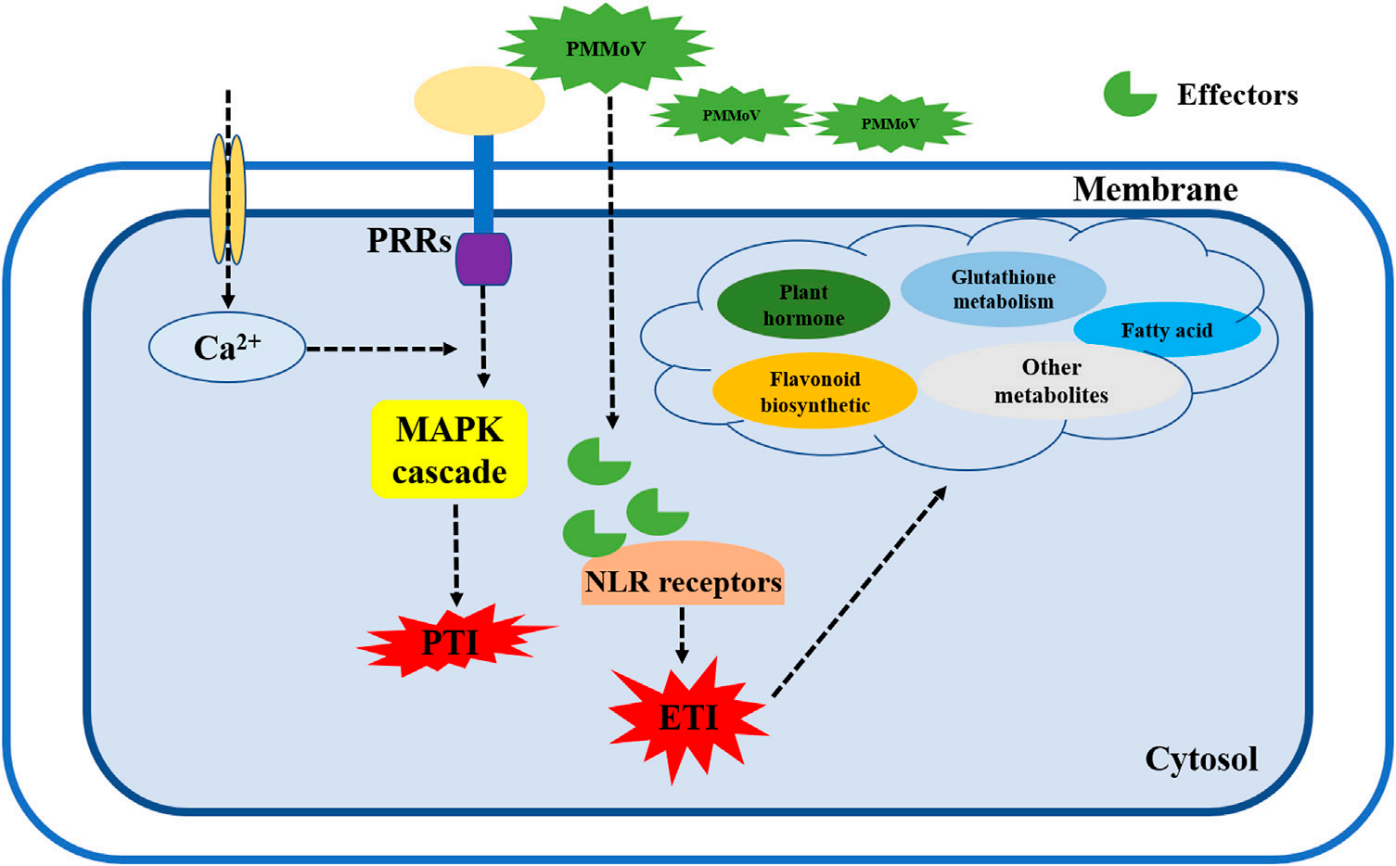
Working model of pepper defense signaling network in response to PMMoV. When the plant membrane receptors sense PMMoV infection, Ca^2+^ and MAPK pathways are activated to induce the first layer of immunity (PTI) in pepper plants. When a viral effector protein breaks through the PTI response, it is bound by NLR-like receptors to stimulate the ETI immune response. At the same time, synthesis of various metabolites such as flavonoids, phytohormones, and fatty acids increases to synergistically defend the plant against further virus attacks. PRRs, pattern recognition receptors; PTI, pattern-triggered immunity; NLR, nucleotide-binding domain and leucine-rich repeat protein; ETI, effector-triggered immunity.

The MAPK signaling pathway is a key pathway by which plants recognize pathogenic bacteria and initiate defense mechanisms. Plants recognize pathogen-associated molecular patterns (PAMPs) through pattern recognition receptors (PRRs), which trigger pattern-triggered immunity (PTI) defense responses to limit survival and reproduction of pathogens. If the pathogen is able to overcome the first line of defense in plant immunity, pathogen effector recognition through nucleotide-binding domain and leucine-rich repeat protein (NLR) receptors will initiate effector-triggered immunity (ETI) (Jones and Dangl, 2006; Bigeard et al., 2015; Yu et al., 2017; Ghosh et al., 2019). We observed significant changes in the expression of genes in the plant–pathogen interaction and MAPK signaling pathways in 17-p63 and 16-217 after PMMoV infection. Most of the related DEGs encoded transcription factors and protein receptors. Two (Capana02g003359 and Capana03g000955) were involved in Ca^2+^ signal transduction, and one (Capana07g001590) was annotated as T459 protein, a tobacco mosaic virus resistance protein. These three DEGs were upregulated in 17-p63 and downregulated in 16-217. By contrast, Capana03g003841, which was predicted to encode a light-dependent short hypocotyls 4-like protein, was downregulated in 17-p63 and upregulated in 16-217. Therefore, we suggest that pepper may recognize PMMoV through PRRs, initiating an immune response to prevent the spread and reproduction of PMMoV. These results also suggest that the tolerant genotype 17-p63 activates PTI through MAPK signaling after sensing PMMoV infection and that PMMoV effector proteins may be able to break through the first layer of the pepper defense mechanism. PMMoV effectors may be recognized by NLR-type pathogen resistance protein(s), activating ETI and inhibiting the replication and spread of PMMoV by promoting synthesis of plant hormones, flavonoids, fatty acids, and other metabolites (Figure 8).

We performed WGCNA analysis of 24 samples and identified two modules, lightcyan1 and blue, that were highly correlated with infected 17-p63 samples at 9 dpi and 16 dpi, respectively. Hub genes associated with infected 17-p63 at 9 dpi (lightcyan1 module) included mainly abiotic stress-related genes. *Capana06g000067* was predicted to encode an F-box-containing protein, a homolog of *Arabidopsis* EDL3 that participates in the abscisic acid signaling pathway and in response to drought stress (Koops et al., 2011). *Capana08g001008* was annotated as desiccation protectant protein Lea14, a stress-related marker gene (Ma et al., 2018), and *Capana05g001996* was predicted to encode glycine-rich cell wall structural protein 1, which has been reported to respond to drought stress (Giarola et al., 2016). *Capana06g000973* was predicted to encode betaine aldehyde dehydrogenase, and *Capana07g002423* was predicted to encode a BTB/POZ domain-containing protein, both of which have been associated with salt stress response in *Arabidopsis* (Missihoun et al., 2015; Wan et al., 2019). In addition to genes related to abiotic stress responses, the hub genes also included *Capana06g000529*, predicted to encode ENHANCED DISEASE RESISTANCE 2-like isoform X1; this was the only lightcyan1 module hub gene previously associated with plant disease resistance. On the basis of the lightcycan1 module hub genes, we speculated that 17-p63 may respond to PMMoV infection at 9 dpi mainly through basal metabolic responses such as changes in hormone metabolism and synthesis of cellular structural proteins. Hub genes associated with infected 17-p63 samples at 16 dpi (blue module) included a number of pathogen resistance-related genes. *Capana01g004093* and *Capana06g000158* were predicted to encode GDSL esterase/lipase, which is essential for systemic resistance to pathogens (Shen et al., 2022). *Capana02g003138* encoded L-ascorbate oxidase, which was reported to activate a variety of defense pathways against cyst nematode in sugar beet (Singh et al., 2020). *Capana06g000648* was predicted to encode aspartic proteinase nepenthesin-2, which can increase plant resistance to pathogens by hydrolyzing their secreted proteins (Simoes and Faro, 2004), and the receptor-like protein kinase THESEUS 1 encoded by *Capana10g000061* positively regulates plant defense responses to *Botrytis cinerea* (Qu et al., 2017). Finally, *Capana00g002203* encodes a subtilisin-like protease, and such proteases play an important part in plant resistance to pathogen infection (Golldack et al., 2003). These results are consistent with a scenario in which PMMoV-infected 17-p63 upregulates pathogen resistance genes at 16 dpi, activating plant immune mechanisms and limiting further PMMoV damage.

## Conclusion

In this study, we analyzed the gene expression profiles of systemic leaves from PMMoV-infected susceptible (16-217) and tolerant (17-p63) pepper genotypes at two stages of infection. PMMoV infection in 16-217 and 17-p63 led to the differential expression of 2959 and 2158 genes, respectively. There were more DEGs in 16-217 than in 17-p63 at 9 dpi, suggesting that the tolerant genotype was less adversely affected by PMMoV early in the infection process. Functional annotation analysis revealed that both genotypes had different expression patterns for DEGs, which were primarily related to plant–pathogen interaction, MAPK signaling, and flavonoid biosynthesis pathways. WGCNA analysis revealed that hub genes associated with infected 17-p63 were mostly related to abiotic stress tolerance at 9 dpi whereas at 16 dpi genes associated with disease resistance was found to be expressed. We identified hub genes that play a central role in resisting PMMoV infection. We concentrated on DEGs in the three pathways above and found that they showed contrasting expression patterns in the two genotypes. We also identified 7 genes that were implicated in disease response. Transcriptome analysis and WGCNA helped us to identify candidate genes that may enable pepper to limit PMMoV infection and propagation, and our findings provide a foundation for further study of the underlying molecular mechanisms of pepper PMMoV resistance. Discovering key resistance genes and understanding the specific mechanisms by which pepper limits PMMoV replication and spread are the subjects of our future work.

## Materials and methods

### Plant growth and virus inoculation

We used *C. annuum* L. genotypes 16-217 (susceptible to PMMoV) and 17-p63 (tolerant). *N. benthamiana* seeds were propagated and stored at the Key Laboratory of Vegetable Germplasm Innovation and Utilization of Hebei, College of Horticulture, Hebei Agricultural University, Baoding, China. All seeds were grown in a growth chamber with a 25°C/16-h light and 22°C/8-h dark regime and 75% relative humidity. The PMMoV-ZJ1 isolate was kindly provided by Dr. Fei Yan (Institute of Plant Virology, Ningbo University, Ningbo, China) and preserved on tobacco plants (Han et al., 2020). For virus inoculation, approximately 0.5 g of PMMoV-infected tobacco leaf tissues were homogenized in phosphate buffer (0.01 M PBS, pH 7.2) at a 1:10 (w/v) ratio. The crude leaf extract was rub-inoculated onto 40-day-old pepper plants. The upper systemic leaves from at least three individual plants were obtained at 1, 3, 5, 7, 9, and 16 dpi, immediately frozen in liquid nitrogen, and stored at −80°C until further use. Leaves of mock-inoculated plants of both genotypes were rub-inoculated with phosphate buffer (0.01 M PBS, pH 7.2) and harvested at the same time points to serve as controls.

### RNA extraction and reverse transcription-polymerase chain reaction (RT-PCR)

Frozen leaf samples were used for RNA extraction. Total RNA was isolated using the Eastep Super Total RNA Extraction Kit (Promega, LS1040, Beijing, China) according to the manufacturer’s instructions. Concentrations and qualities of the isolated RNA samples were monitored using a NanoDrop 2000 spectrophotometer (Thermo Fisher Scientific, Waltham, MA, United States). First-strand cDNA was synthesized from 1 μg of total RNA with the PrimeScript RT Reagent Kit (Perfect Real Time) (Takara Bio Inc., China) according to the manufacturer’s instructions. PCR amplification was performed in a final volume of 50 μl that contained 5 μL of 10× PCR buffer (Mg^2+^ Plus), 4 μL dNTP mixture (2.5 mM each), 0.25 μL Taq (Takara, Shiga, Japan), 1 μL forward primer, 1 μL reverse primer, 2.5 μL cDNA, and 36.25 μL double-distilled H_2_O. PCR reaction conditions were 95°C for 5 min; 35 cycles of 95°C for 30 s, 55°C for 30 s, and 72°C for 30 s; 72°C for 7 min; and a final hold at 4°C. The primers used in this experiment are summarized in Supplementary Table S5.

### RNA sequencing

We pooled the upper young leaves from at least three individual plants to serve as one sample, and there were three replicate samples for each combination of genotype, treatment, and timepoint. mRNA was purified from extracted total RNA (5 μg) using Dynabeads Oligo (dT) (Thermo Fisher, CA, United States) with two rounds of purification. Following purification, the mRNA was fragmented into short fragments using divalent cations under elevated temperature (Magnesium RNA Fragmentation Module, NEB, e6150, United States) at 94°C for 5–7 min. The cleaved RNA fragments were reverse-transcribed to create cDNA using SuperScript II Reverse Transcriptase (Invitrogen, 1896649, United States), and cDNA was used to synthesize U-labeled second-strand DNA with *E. coli* DNA polymerase I (NEB, m0209, United States), RNase H (NEB, m0297, United States), and dUTP Solution (Thermo Fisher, R0133, United States). An A base was then added to the blunt ends of all strands, preparing them for ligation to the indexed adapters. Each adapter contained a T-base overhang to enable ligation to the A-tailed fragmented DNA. Dual-index adapters were ligated to the fragments, and size selection was performed using AMPure XP beads. After treatment of the U-labeled second-strand DNA with heat-labile UDG enzyme (NEB, m0280, United States), the ligated products were amplified under the following PCR conditions: initial denaturation at 95°C for 3 min; 8 cycles of denaturation at 98°C for 15 s, annealing at 60°C for 15 s, and extension at 72°C for 30 s; and a final extension at 72°C for 5 min. The average insert size for the final cDNA libraries was 300 ± 50 bp. We performed the 2-bp × 150-bp paired-end sequencing on the Illumina NovaSeq 6000 platform (LC-Bio Technology Co., Ltd., Hangzhou, China) following the vendor’s recommended protocol.

### DEGs and GO and KEGG enrichment analysis

DEGs were identified between two different groups using DESeq2 software (Love et al., 2014) based on a *p*-value < 0.05 and an absolute fold change ≥2. Venn diagrams, volcano plots, and heatmaps were drawn using OmicStudio tools at https://www.omicstudio.cn/tool. All DEGs were mapped to GO terms in the Gene Ontology database (http://www.geneontology.org/), and the number of genes associated with each term was calculated. Significantly enriched GO terms in DEG sets compared with the genome background were identified using hypergeometric tests (*p* < 0.05). DEGs were also assigned to pathways in the KEGG database (http://www.genome.jp/kegg), and significantly enriched KEGG pathways were identified (Kanehisa et al., 2021).

### WGCNA network analysis

The WGCNA R package was used to construct co-expression networks (Langfelder and Horvath, 2008). Before performing WGCNA analysis, we removed genes with no or very low expression (FPKM < 0.5) in all samples. We used the pickSoftThreshold tool in the WGCNA package to select an appropriate soft thresholding power. Modules were defined as clusters of densely interconnected genes. Topological overlap measures were used to construct network interconnectedness, and genes were hierarchically clustered based on topological matrix similarity (Li and Horvath, 2006). Modules are defined as clusters of highly interconnected genes. We calculated the connectivity of each gene as the sum of its connection strengths with other network genes and used intramodular connectivity to identify the most highly connected hub gene within a module as the module representative. Connectivity of a gene within a module represents the regulatory relationship of this gene with other genes, and a high connectivity value suggests that a gene plays an important role in a module. We selected the top 10 genes of each module based on their connectivity values as candidate genes to build a gene interaction network (Zhu et al., 2019). Cytoscape 3.7 software (https://cytoscape.org) was used to visualize the regulatory relationships among genes in modules.

### qRT-PCR analysis

To validate the RNA-seq results, we selected eight DEGs and measured their expression by qRT-PCR. One microgram of total RNA per sample was used for cDNA synthesis with the PrimeScript 1st Strand cDNA Synthesis Kit (Takara, Dalian, China) according to the manufacturer’s instructions. The 20-µL qRT-PCR reactions contained ChamQ Universal SYBR qPCR Master Mix (2×) (Vazyme, Nanjing, China), 0.4 μL each of forward and reverse primers, 2 μL cDNA template, and 7.2 μL double-distilled H_2_O. The qRT-PCR reactions (95°C, 30 s; 95°C, 10 s; 60°C, 30 s; 40 cycles) were performed using the SYBR Green method on a CFX96 Touch Real-Time PCR Detection System. We used the beta tubulin gene (Gene ID: 107866876) as the internal control gene (Han et al., 2020), and three biological replicates were performed for each experiment. The primers were designed according to gene sequences from the *C. annuum* L_Zunla-1_Release_2.0 database (http://peppersequence.genomics.cn) and synthesized by Sangon Biotech, Shanghai, China (Supplementary Table S5). We used the 2^−ΔΔCT^ method to quantify relative gene expression (Schmittgen and Livak, 2008).

## Data Availability

The original contributions presented in the study are publicly available. This data can be found here: https://www.ncbi.nlm.nih.gov/. Accession number: PRJNA935813.

## Supplementary material

SUPPLEMENTARY FIGURE S1 Symptoms of Pepper mild mottle virus (PMMoV) infection in upper systemic leaves of 16-217 at 16 dpi. Mock represents mock-inoculated plant leaves. PMMoV represents PMMoV-inoculated plant leaves (bar = 1 cm).

SUPPLEMENTARY FIGURE S2 Semiquantitative RT-PCR for the PMMoV coat protein gene in the upper systemic leaves. **(A)** The result of semiquantitative RT-PCR detection in 16-217. **(B)** The result of semiquantitative RT-PCR detection in 17-p63. M: DL2000 marker; 1 and 2: upper systemic leaves at 1 dpi; 3 and 4: upper systemic leaves at 3 dpi; 5 and 6: upper systemic leaves at 5 dpi; 7 and 8: upper systemic leaves at 7 dpi. 9 and 10: upper systemic leaves at 9 dpi; 11 and 12: upper systemic leaves at 13 dpi. Each sample type includes three biological replicates.
