# Supplementary figures and images for: Transcriptome analysis of two pepper genotypes infected with pepper mild mottle virus
Source: Front Genet. 2023 Apr 20;14:1164730. doi: 10.3389/fgene.2023.1164730 (PMC10156976; doi:10.3389/fgene.2023.1164730)

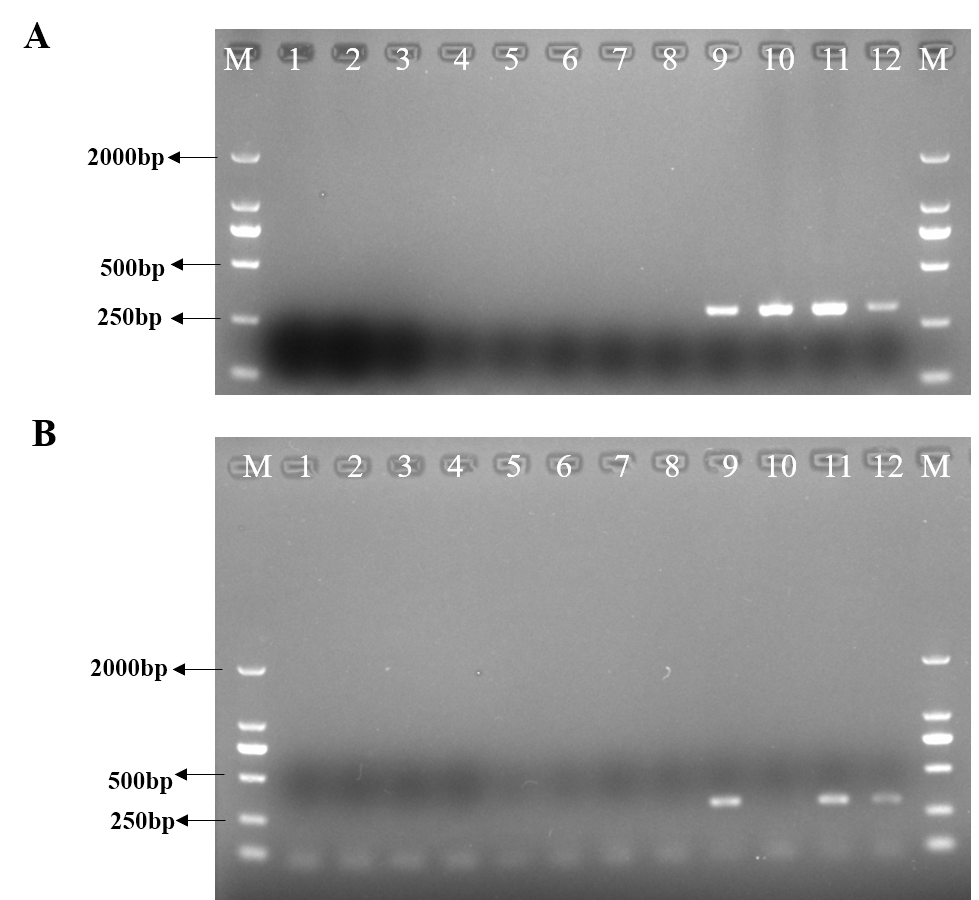

Supplement: Supplementary file 3 [file Image2.TIF]

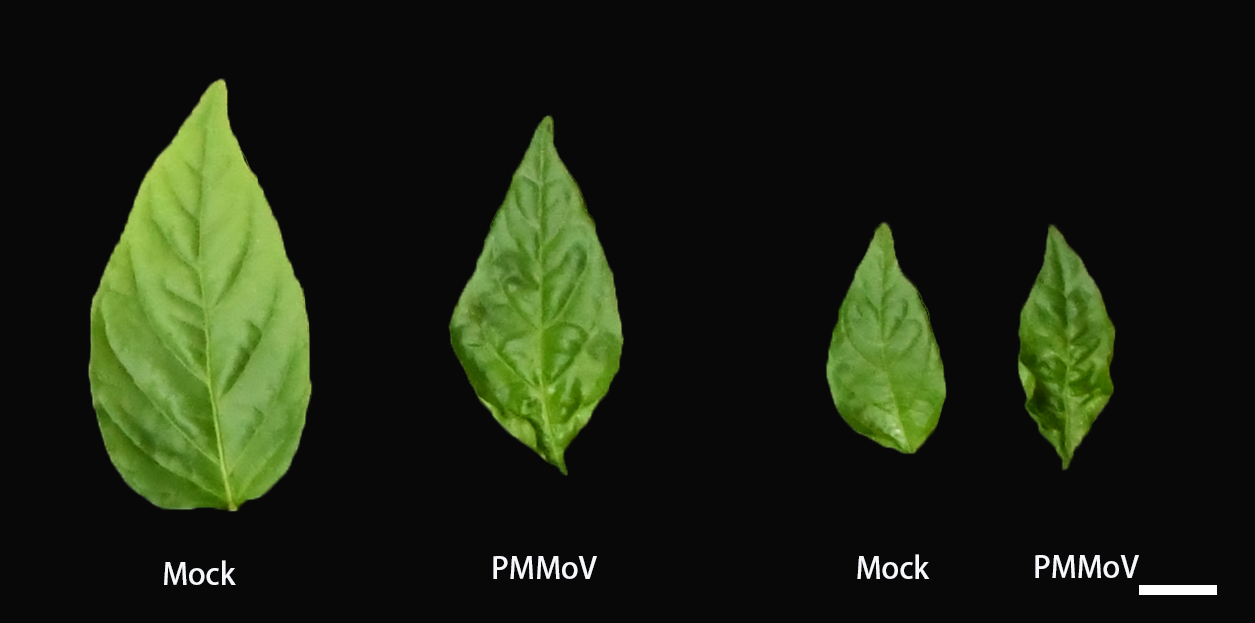

Supplement: Supplementary file 6 [file Image1.PNG]
